# Editorial: Adaptation mechanisms of grass and forage plants to stressful environments, volume II

**DOI:** 10.3389/fpls.2023.1323841

**Published:** 2023-11-23

**Authors:** Jing Zhang, Yu Chen, Mao-Feng Chai, Sergey Shabala, Ke-Hua Wang, Jin-Lin Zhang

**Affiliations:** ^1^ College of Agro-grassland Science, Nanjing Agricultural University, Nanjing, China; ^2^ Key Laboratory of National Forestry and Grassland Administration on Grassland Resources and Ecology in the Yellow River Delta, College of Grassland Science, Qingdao Agricultural University, Qingdao, China; ^3^ Tasmanian Institute of Agriculture, University of Tasmania, Hobart, TAS, Australia; ^4^ Department of Turfgrass Science and Engineering, College of Grassland Science and Technology, China Agricultural University, Beijing, China; ^5^ State Key Laboratory of Herbage Improvement and Grassland Agro-ecosystems, Key Laboratory of Grassland Livestock Industry Innovation, Ministry of Agriculture and Rural Affairs; College of Pastoral Agriculture Science and Technology, Lanzhou University, Lanzhou, China

**Keywords:** grass and forage plants, stress tolerance, gene functional characterization, organic acid, host-microbe interactions, nutrient uptake

## 1 Introduction

In nature, plants encounter various challenges from stressful environments, including high and low temperatures, drought, salinity, heavy metals, and nutrient deficiency, which adversely affect plant growth and development (Zhang et al., 2022). It is well-established that global warming is occurring, leading to frequent and extreme climate fluctuations, resulting in frequent natural disasters and environmental problems. Additionally, the increasing global population presents greater challenges for food and ecological security (Gupta et al., 2020). Therefore, cultivating more stress-resistant crop varieties and increasing crop yield and quality are essential for addressing global food security and sustainable development issues.

Grass and forage plants with relatively more powerful stress resistance fulfill numerous functions, including beautifying landscapes, protecting the environment, enhancing recreational activities, and providing fodder for livestock and wildlife (Huang, 2021). In comparison to crops, grass and forage plants display a diverse range of breeding mechanisms, including cross-pollination, self-pollination, and hybridization between different species. This diversity contributes to a wide genetic variation within these plants and enables them to thrive in more stressful environments (Huang, 2021). Therefore, there is an imperative necessity to investigate the underlying stress-tolerant mechanisms that might feedback grass, forage and crop plant breeding for stress tolerance improvement.

Despite significant advances in understanding the mechanisms of grass and forage plants to stressful environments, there remain knowledge gaps in these areas, and this Research Topic aims to address these gaps. In this Research Topic, 11 articles written by 75 researchers were published.

## 2 Functional characterization of genes relevant to stress tolerance

The Full-length cDNA overexpression (FOX) system is a molecular biology technique used for gene mining by overexpressing full-length complementary DNA (cDNA) in cells or organisms (Ichikawa et al., 2006). The FOX hunting system has been successfully applied for stress-tolerant genes screening in *Arabidopsis* and rice. For example, *TsHsfA1d* and *OsREX1-S* identified *via* FOX hunting system functioned as positive regulators of heat stress and cadmium stress, respectively (Higashi et al., 2013; Kunihiro et al., 2014). In this Research Topic, Zheng et al. identified eleven salt-tolerant genes using FOX hunting system in *Zoysia matrella*. They particularly focused on a novel salt-inducible candidate gene called *ZmGnTL*. Their findings revealed that *ZmGnTL* improves salt tolerance by regulating ion homeostasis, scavenging reactive oxygen species, and adjusting osmotic balance.

High-affinity K^+^ transporters (HKTs) are transmembrane cation transporters that play a pivotal role in Na^+^ or Na^+^-K^+^ co-transport, thereby regulating salt tolerance in plants (Horie et al., 2007). Therefore, *HKTs* represent valuable gene resources for enhancing plant salt tolerance. In this Research Topic, Haxim et al. characterized a novel *HKT* gene named *SeHKT1;2* from a halophyte *Salicornia europaea*. SeHKT1;2 selectively transports Na^+^ rather than K^+^ and is an important target for understanding the mechanisms of salt tolerance in plants.

Plant plasma membranes (PMs) serve important functions in maintaining intracellular stability and exchanging information with the external environment. Studying the dynamics of the PM proteome is crucial for elucidating cellular regulation in response to various stimuli. However, analyzing the PM proteome poses challenges due to the low abundance of PM proteins in the total cellular protein pool (Chen and Weckwerth, 2020). To enhance the separation and enrichment of PM proteins, Yang et al. developed a simplified method that combines differential centrifugation and Brij-58 treatment. This method increased the abundance of PM proteins in the enriched fraction while reducing contamination from other organellar proteins.

## 3 Regulations of natural metabolites or synthetic chemicals on stress tolerance

The remediation of saline-alkali and heavy metal-contaminated lands plays a crucial role in protecting the ecological environment, improving land availability, and promoting sustainable agriculture (Khan and Bhatt, 2023). Chemical substances, such as organic acids and biochar, have the potential to react with pollutants, neutralizing or transforming them into less harmful forms. In this Research Topic, Yang et al. discovered that the exogenous application of citric acid reduced soil salinity and increased soil nutrient content, root vigor, and photosynthesis in sweet sorghum. As a result, the stress tolerance of sweet sorghum was improved, leading to higher biomass yield. Additionally, Meng et al. found that the use of 5% corn straw biochar effectively alleviated the toxicity of Pb to red clover and the associated soil. Above findings contribute to sustainable development and foster the harmonious coexistence between humans and nature.

## 4 Omics-related studies

Recent significant progress in omics techniques, such as transcriptomics, genomics, proteomics, and metabolomics, has significantly contributed to the profound understanding of the molecular mechanisms underlying plant stress tolerance (Singhal et al., 2021). In this Research Topic, Wang et al. found that application of P fertilizer resulted in improved root structure and increased levels of soluble sugar and soluble protein. The integration of the transcriptome and metabolome revealed the impact of P on the biosynthesis of N-acetyl-L-phenylalanine, L-serine, lactose, and isocitrate during the cold acclimation period.

Strong ultraviolet radiation and low temperature environments can induce the synthesis of specific secondary metabolites in plants as a defense mechanism against severe environmental stresses. To understand the adaptive mechanism of *Draba oreades Schrenk* at high altitude, Lei et al. conducted a comprehensive evaluation of the metabolome in plants at 3800 m, 4000 m, and 4200 m, respectively. Based on the metabolome data, ten crucial metabolites were identified as potential biomarkers. The levels of L-phenylalanine, L-histidine, naringenin-7-O-Rutinoside-4’-O-glucoside, and apigenin, which are associated with flavonoid biosynthesis and plant disease resistance, increased with the increase of altitude.

## 5 Cross stress tolerance

In natural environments, plants frequently encounter various simultaneous stresses, leading to the occurrence of cross-tolerance phenomena (Zhang et al., 2023). Climate change exposes plants to multiple abiotic stresses simultaneously. While the responses of plants to individual stresses have been extensively studied, it is challenging to speculate and infer the effects of stress combinations based on solely these studies. In this Research Topic, Zhou et al. examined the responding mechanism of bermudagrass to combined low temperature and salt treatments. It was observed that low temperature treatment reduced the relative growth rate, chlorophyll fluorescence transient curve, biomass, and crude fat content. Conversely, mild salt addition alleviated cold stress-induced damage by enhancing photosynthesis and improving the enzymatic activity of antioxidant. This study provides a comprehensive understanding of the probable interaction mechanism between low temperature and salt stress in grass plants, offering valuable insights for fodder growth in cold regions.

## 6 Future research

The articles presented in this Research Topic make a significant contribution to addressing gaps in our understanding of the role of complex signaling transduction pathways in grass and forage plants in response to various stressful environments. These articles also highlight the identification of stress-tolerant genes, beneficial natural metabolites, and root-associated microbes, which serve as valuable resources not only for grass and forage plants, but also for other crops. CRISPR/Cas is a valuable biotechnological approach for breeding crops with increased tolerance to stressful environments (Zhu et al., 2020), while no CRISPR/Cas-related research has been included in this Research Topic. We look forward to more articles to be published exploring the application of CRISPR/Cas and further advancements in creating stress-tolerant grass and forage germplasms without transgenic elements.

## Author contributions

JZ: Funding acquisition, Supervision, Writing – original draft, Writing – review & editing. YC: Funding acquisition, Writing – review & editing. M-FC: Writing – review & editing. SS: Writing – review & editing. K-HW: Writing – review & editing. J-LZ: Writing – original draft, Writing – review & editing.
